# Mercury and selenium concentrations in fishes of the Upper Colorado River Basin, southwestern United States: A retrospective assessment

**DOI:** 10.1371/journal.pone.0226824

**Published:** 2020-01-13

**Authors:** Natalie K. Day, Travis S. Schmidt, James J. Roberts, Barbara C. Osmundson, James J. Willacker, Collin A. Eagles-Smith

**Affiliations:** 1 U.S. Geological Survey, Southwest Biological Science Center, Moab, Utah, United States of America; 2 U.S. Geological Survey, Colorado Water Science Center, Fort Collins, Colorado, United States of America; 3 U.S. Fish and Wildlife Service, Grand Junction, Colorado, United States of America; 4 U.S. Geological Survey, Forest and Rangeland Ecosystem Science Center, Corvallis, Oregon, United States of America; University of Arkansas Fayetteville, UNITED STATES

## Abstract

Mercury (Hg) and selenium (Se) are contaminants of concern for fish in the Upper Colorado River Basin (UCRB). We explored Hg and Se in fish tissues (2,324 individuals) collected over 50 years (1962–2011) from the UCRB. Samples include native and non-native fish collected from lotic waterbodies spanning 7 major tributaries to the Colorado River. There was little variation of total mercury (THg) in fish assemblages basin-wide and only 13% (272/1959) of individual fish samples exceeded the fish health benchmark (0.27 μg THg/g ww). Most THg exceedances were observed in the White-Yampa tributary whereas the San Juan had the lowest mean THg concentration. Risks associated with THg are species specific with exceedances dominated by Colorado Pikeminnow (mean = 0.38 and standard error ± 0.08 μg THg/g ww) and Roundtail Chub (0.24 ± 0.06 μg THg/g ww). For Se, 48% (827/1720) of all individuals exceeded the fish health benchmark (5.1 μg Se/g dw). The Gunnison river had the most individual exceedances of the Se benchmark (74%) whereas the Dirty Devil had the fewest. We identified that species of management concern accumulate THg and Se to levels above risk thresholds and that fishes of the White-Yampa (THg) and Gunnison (Se) rivers are at the greatest risk in the UCRB.

## Introduction

Mercury (Hg) and selenium (Se) are ubiquitous contaminants affecting freshwater environments on a global scale [1, 2]. Inorganic Hg is introduced into the environment from human activities such as mining and atmospheric pollution derived from fossil fuel combustion [1, 3–5]. When reactive forms of inorganic Hg are transported to aquatic habitats, microbial conversion into methylmercury (MeHg) greatly increases its bioavailability, toxicity, and bioaccumulation potential [6]. Selenium is an essential micronutrient required for normal growth and development [7] but human activities, including power generation, oil refining, mining, and irrigation drainage [2] have dramatically increased concentrations in many aquatic environments [8] including in the Upper Colorado River Basin (UCRB).

**Fig 1:**
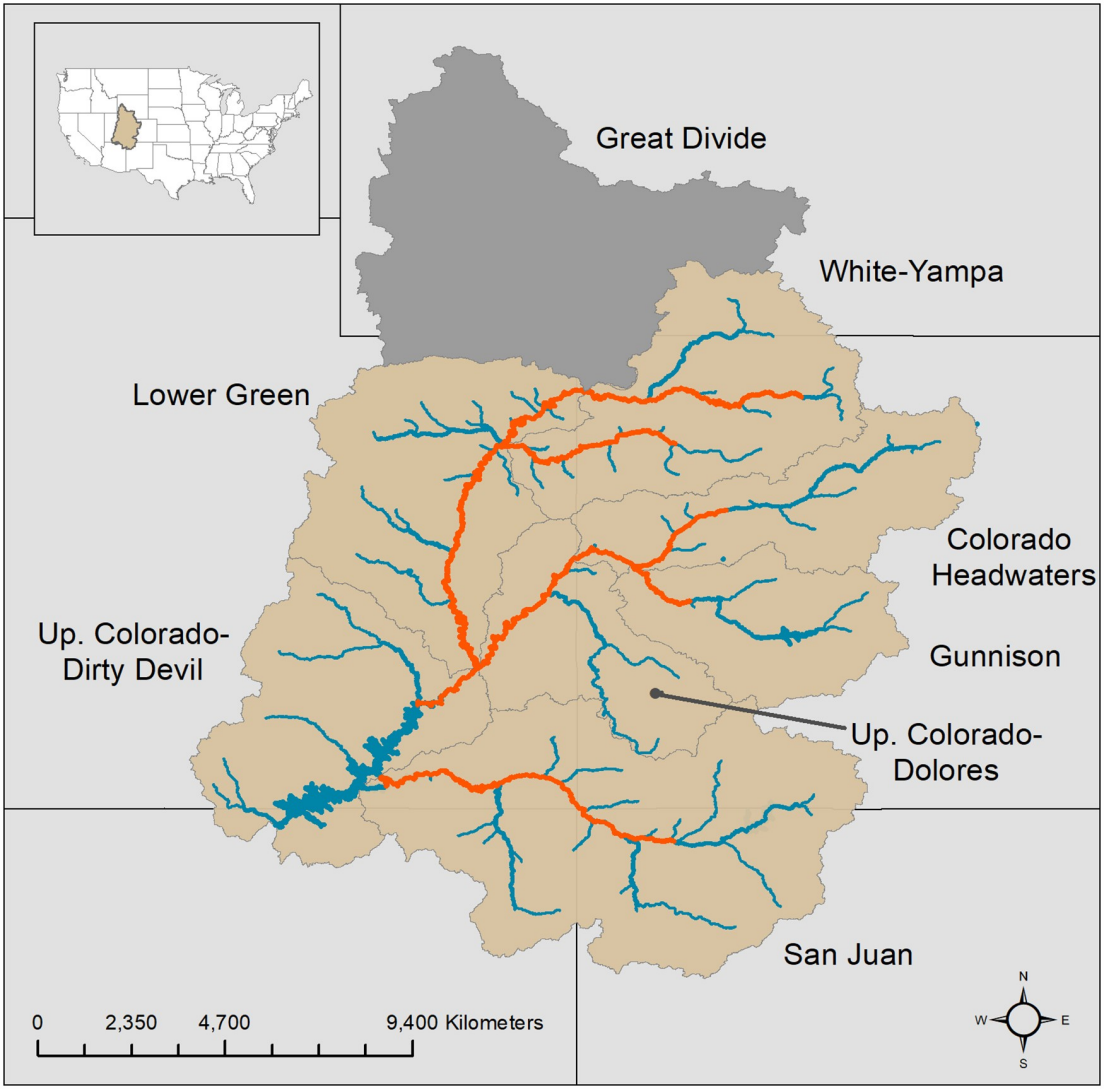
The Upper Colorado River Basin spans 5 states and is comprised of 8 tributaries, including the Colorado Headwaters, Gunnison, Upper Colorado-Dolores, White-Yampa, Lower Green, Upper Colorado-Dirty Devil, and the San Juan. We did not include data within the Great Divide- Upper Green tributary (shaded grey) above Flaming Gorge Dam as it is disconnected from the rest of the UCRB. River shaded orange is critical habitat for the federally endangered Colorado Pikeminnow.

Ecological risks associated with accumulation of Hg and Se may be particularly acute in Western North America due to a myriad of land use, habitat, and species composition changes [9]. The UCRB (Fig 1) is a major component of this region, draining about 10% of the land area (293,000 km^2^) of the 11 most western United States, with substantial socio-political importance [10] owing to wide-spread modification of streamflow to provide electricity and water to communities within and outside of the UCRB watershed. Beyond land and water management practices and infrastructure, native fish assemblages within the UCRB are also threatened from a suite of invasive fishes that are becoming more widespread in this region [11]. Invasive species (including fish, invertebrates, and plants) and hydrologic alteration have been identified as two of the four agents of global change that can influence the magnitude of Hg risk [12]. Thus, fisheries and water managers addressing these issues are also increasingly concerned that Hg and Se contamination may now also be a threat to endangered fishes in the UCRB [13, 14].

The imperiled native fish assemblage of the UCRB [15, 16] includes four species listed as endangered under US Endangered Species Act (ESA) including the Bonytail (*Gila elegans*), Colorado Pikeminnow (*Ptychocheilus lucius*), Humpback Chub (*Gila cypha*), and Razorback Sucker (*Xyrauchen texanus*). These fishes have unique life histories that include long-distance annual migrations (e.g., Colorado Pikeminnow) [17, 18] including movements both within and among major tributaries of the UCRB. Some of these fishes are also highly piscivorous (Colorado Pikeminnow) and all are long-lived, making them particularly susceptible to Hg and Se accumulation.

Mercury readily biomagnifies through food webs and confers no biological benefit [19], whereas Se is an essential element required for many biological functions (e.g., reproduction) that accumulates in organisms, but generally does not biomagnify through food webs [20, 21]. Both Hg and Se elicit toxic effects at concentrations frequently observed in the environment [22, 23], but when they co-occur they can interact in complex ways, including Se potentially ameliorating some Hg toxicity in fish [24, 25]. Thus, it can be informative to understand the relative exposure of these two contaminants together. Due to the differences in environmental conditions (e.g., water quality, flow, habitat) [1, 20], sources of Hg and Se, and species composition throughout the UCRB [21, 26, 27], it is expected that risks to fish might vary across this expansive riverscape.

While invasive species, habitat degredation, and flow alterations are important stressors to the fish assemblages in the UCRB, less attention has been given to contaminants as possible stressors. However, both Hg and Se are included in population recovery plans for some managed species of the UCRB as possible stressors limiting population growth [14]. Over the past 50 years, a series of small studies have sampled for Hg and Se in various fish species but rarely were water, sediments, or food web items paired with fish tissue samples making it difficult to explore controls on exposure and accumulation. Rather, there is a large dataset of temporally and spatially explicit fish tissue samples that can be explored to identify which species are most vulnerable to accumulate Hg and Se and if accumulation of these elements varies spatially. Owing to the differences in environmental conditions (e.g., flow, water quality, sources of mercury and selenium) and species composition, among the major tributaries of the UCRB, we expect the concentrations of Hg and Se in fish tissue might vary across the riverscape. These spatial variations may have important conservation implications since UCRB fish management plans are based on major tributaries [13, 14, 17, 28–30].

In this paper, we employed linear mixed effects models (LMEM) to retrospectively assess THg (total mercury) and Se in fish tissues of the UCRB. First, we estimate average THg and Se in fish species of the UCRB while accounting for fish size, sampling location, and sampling year. Secondly, we characterize spatial patterns of THg and Se concentrations in fish assemblages of the UCRB while accounting for the effects of fish size, sampling location, species and sampling year. Finally, we characterize the co-accumulation of THg and Se (Se:Hg molar ratios) in species and among major tributaries of the UCRB. To our knowledge, this is the first comprehensive basinwide assessment of THg and Se accumulation into the fishes of the UCRB.

## Methods

### Study area

The UCRB extends across the southwestern US inclusive of the states of Wyoming, Utah, Colorado, Arizona, and New Mexico. The UCRB is comprised of 8 major tributaries, as designated by the U.S. Geological Survey four-digit hydrologic unit codes (HUC), including the Colorado Headwaters, Gunnison, Upper Colorado-Dolores, Great Divide-Upper Green, White-Yampa, Lower Green, Upper Colorado-Dirty Devil, and the San Juan (Fig 1). Hydrographs of UCRB streams and rivers are dominated by snowmelt runoff in the headwater regions, which usually begins in late April, has a highly variable peak flow (based on the snowpack in any given year) and has finished receding by mid- to late July. A large portion of the basin is comprised of semiarid or arid plains that do not contribute substantially to stream flow but may contribute substantial amounts of sediment to the system, especially during monsoonal rain events.

### Data compilation

We compiled THg and Se concentration data for fish tissues collected from river ecosystems throughout the UCRB (HUC-14; Upper Colorado) from Federal and State databases. Eagles-Smith et al. [9] describes the details on data quality, data validation, assumptions, and standardization from these Federal and State data sources. We augmented these 1,861 individual fish records with another 463 records from several sources not included in the original database [31–34]. The cumulative dataset spans from 1962 to 2011 and includes 2,324 individual fish records (1,355 with both THg and Se concentrations, 604 with only THg concentrations, and 365 with only Se concentrations) from 33 species of fish [35]. There is no age information in the database because non-lethal sampling methods are required to sample endangered and threatened species in the Upper Colorado River Basin or the existing sampling design employed during the time of collection did not include methods for aging. We used spatial coordinates from each fish tissue sample and assigned it a four-digit HUC code to assign each sample to one of the seven major tributaries. A subset of samples (n = 70) did not have specific location information, but rather the beginning and ending coordinates of the reach (average length of 89 km) from which they were collected. For these sites, we aggregated samples to a site location at the mid-point of each reach from which they were collected. For modeling purposes, each fish sample was aggregated to one unique sample location every 10-linear river km. We did not include data within the Great Divide-Upper Green tributary above Flaming Gorge Dam as it is disconnected from the rest of the UCRB. The remaining number of fish samples located within the Great Divide-Upper Green tributary below Flaming Gorge Dam were few and combined with the Lower Green tributary to enable more robust spatial analysis. Thus, our analysis considered data from seven UCRB tributaries (i.e., four-digit HUC basins).

To convert whole-body THg concentrations to muscle tissue concentrations we divided by 0.74, the average ratio of whole-body to muscle concentration [36, 37]. We used species and family specific conversion values to convert whole-body Se concentrations to muscle Se concentrations (S1 Table) [38]. We did not differentiate between skin-on muscle concentrations and skinless muscle concentrations because the difference between these sample types is typically small (<10%) [39]. We converted between wet-weight concentrations and dry-weight concentrations using the original moisture content data when available or the mean tissue-specific moisture content from the original dataset (76%). We report all THg concentrations in μg/g wet-weight muscle acknowledging that > 90% of the THg is likely methylmercury [19]. All Se concentrations are reported in μg/g dry-weight muscle.

We evaluated hazards to fish health by comparing THg and Se in fish tissues with benchmark concentrations associated with sub-lethal effects in fishes (i.e., growth, reproduction, development, behavior; 0.2 μg THg/g wet-weight whole body and 4.0 μg Se/g dry-weight whole body [40, 41]). We choose to use these benchmarks because much of the waters included in the UCRB are designated Critical Habitats by the U.S. Fish and Wildlife Service. Critical Habitats are geographic areas that contain endangered species at the time of listing, and these areas are managed to minimize hazards to endangered fishes of the UCRB. Thus, sub-lethal benchmarks (i.e., Hg benchmark used here) and benchmarks developed specifically for the managed species (i.e., Se benchmark used here) are preferred over more generic values. These benchmarks were converted from whole-body to muscle concentrations to match our converted database values. For THg we divided the whole-body fish health benchmark by 0.74 (see above) to derive 0.27 μg THg/g wet-weight muscle, heretoafter called the THg-benchmark concentration. For Se, the whole body fish health benchmark was derived by the US EPA to be protective of fish in general, and was converted to a muscle based threshold by multiplying the fish health standard (see above) by 1.27, the correction factor employed by US EPA to translate the criterion values between whole-body and muscle [38]. We refer to this value as the Se-benchmark concentration (5.1 μg Se/g dry-weight muscle) throughout the paper. These benchmarks (Hg and Se) are used to gage species specific frequency of exceedances and not for use as an indicator of species-specific sensitivities to each benchmark.

### Modeling approach

We used linear mixed effect models to estimate the amount of THg and Se in different fish species and fish assemblages across the UCRB. Due to the nature of our dataset (i.e., many fish species collected across different regions at different sites during different time periods), using LMEM allowed for an estimation of means while also taking in account potential sources of variation. A simple linear regression of THg concentration and fish species would provide an average THg concentration for all fish species; however, it is likely that the relationship is different among subregions or during different time periods. Thus, when comparing concentrations of THg or Se in species or of assemblages, accounting for these major factors can provide a more informed estimate of means. Within a LMEM, fixed effects are the main, reproducible factors of interest (i.e., species or fish assemblage) and a mean is estimated for each level of the factor. Much of the variance associated with those means can be attributed to some other factors (e.g., time of sampling, types of species found in the assemblage, fish sizes). Thus, including these factors as random effects in the model can help inform properties of the fixed effects and explain much of the variation in the mean.

### Size-standardization

We normalized individual THg concentrations by fish length following Eagles-Smith et al. [42] to facilitate comparisons across individuals of different sizes. Of the 1,959 fish records with THg concentrations, only 932 were associated with length measurements (mm). Although no age assessments were made of fish because it was not part of the various study foci, a range of sample lengths were sampled suggestive that a range of age classes were targeted in sampling (S2 Table). The number of observations for some species limited our ability to develop a model to size-adjust THg concentrations. Therefore, related species with similar ecology (i.e., feeding traits and life history) and phylogeny (e.g., salmonids) were grouped into aggregate species groups to develop a model that size-adjusted THg tissue concentrations (S2 Table). To model size-normalized Hg concentrations, we then constructed LMEM for each species group that included total length as a fixed covariate and site nested in tributary and a species × total length interaction as random effects. Using this model we predicted each fish’s THg concentration at the median total length of each species, and then added residuals from each fish back to the modeled estimate in order to propagate individual variation through the process [42]. Including a species × total length interaction as a random effect allowed us to account for variation among species within an aggregate group while pooling variance among species within a group to allow estimation of the THg-size relationship in underrepresented species. We did not include the species × total length interaction if the aggregate group was comprised of only one species (i.e., Colorado Pikeminnow). We did not size correct aggregate species groups when Akaike Information Criterion (AIC) suggested that the inclusion of length did not improve the model. We also did not size correct aggregate species groups if there were fewer than 25 individuals or if individuals were found in fewer than 3 tributaries. Therefore, for those species that could not be size-adjusted (Razorback Sucker, Bluehead Sucker (*Catostomus discobolus*), Black Bullhead (*Ameiurus melas*), Mountain Sucker (*Catostomus platyrhynchus*), Longnose Sucker (*Catostomus Catostomus*)), we present raw THg concentrations and do not include them in any models with size-adjusted fish. We did not size standardize Se concentrations because Se concentrations are generally not correlated with fish size [43].

### Quantifying differences in THg and Se among species and fish assemblages

We assessed the overall patterns of THg and Se concentrations in fish species and fish assemblages using multiple statistical approaches. Initially, we evaluated the potential risk of individual fish species and fish assemblages to THg and Se using fish health benchmarks. We used raw data (i.e., not size corrected) to calculate geometric mean THg and Se concentrations for each species individually as well as the geometric mean concentrations of fish assemblages found in each tributary. We used these descriptive means to evaluate the frequency of exceedance of fish health benchmarks by species and tributary.

In subsequent analyses, we used linear mixed effects models to calculate species mean concentrations of THg and Se while accounting for spatial, size, and temporal sample biases. Least-squares means for THg and Se concentrations in fish species were derived by using size-adjusted THg or Se (not size-adjusted) concentrations as the dependent variables, species as a fixed variable, and site nested in tributary and year as random effects. Nesting site in tributary accounts for spatial variation within a tributary, as tributaries cover large areas and can themselves have spatial variation in THg and Se exposure. Because not all species occurred evenly among tributaries, but we wanted to compare THg and Se among tributaries, we utilized a similar approach as described above to characterize THg and Se in fish assemblages among tributaries. For this model, we used size-adjusted THg or Se as dependent variables, site nested in tributary as a fixed variable, and species and year as random effects. Pairwise comparisons were made as described below (statistical approach section).

Finally, because Colorado Pikeminnow are managed as three different populations (Green River, Colorado River, San Juan River) [30] with population estimates calculated regularly for ≥450 mm adults, we estimated the average THg and Se in adult sized Colorado Pikeminnow for each population. Data for Colorado Pikeminnow in each tributary were aggregated by combining the tributaries associated with each population (Green River population = White-Yampa + Lower Green tributaries, the Colorado population = Colorado Headwaters + Upper Colorado-Dirty Devil + Upper Colorado-Dolores + Gunnison tributaries, and the San Juan population included just the San Juan tributary). We used a linear mixed effects model to predict THg and Se concentrations for Colorado Pikeminnow 450 mm in length with THg and Se (neither size-adjusted) as dependent variables, total length and a total length by site interaction were nested in population as fixed effects, and year was treated as a random effect. The interaction term as a fixed effect allows for the estimated mean concentration of a 450 mm-long Colorado Pikeminnow to vary by location within population.

### Se:Hg molar ratios in fish tissue

We characterized Se:Hg molar ratios for each species using linear mixed effect models as described above. We converted Se to wet-weight concentrations using the original moisture content data when available or the mean tissue-specific moisture content from the original dataset (76%). We divided Se and Hg concentrations by their respective molecular weights to obtain a molar mass and calculated a Se:Hg molar ratio for each individual fish. We included species as a fixed effect, site nested in tributary, and year as random effects. Finally, we assessed Se:Hg molar ratios of fish assemblages by tributary by including site nested in tributary as a fixed effect, and species and year as random effects.

### Statistical approach

Unless otherwise specified, all THg and Se concentrations were log (base 10) transformed prior to analysis to meet assumptions of heteroscedasticity and normality of residuals. We calculated degrees of freedom and the F-value using the Kenward-Roger’s approximation [44]. We back transformed model estimates to linear space and standard errors were estimated with the delta method [45]. Pairwise comparisons were made among estimated marginal means among species and assemblages using α = 0.05 with a tukey’s multiplicity adjustment. All statistics were performed in R software version 3.3.2 utilizing the following packages: MuMin version 1.15.6, lmerTest version 2.0–33, and lsmeans 2.30.0 (R Development Core Team) [46].

## Results

### THg and Se concentrations in fish species

**Fig 2:**
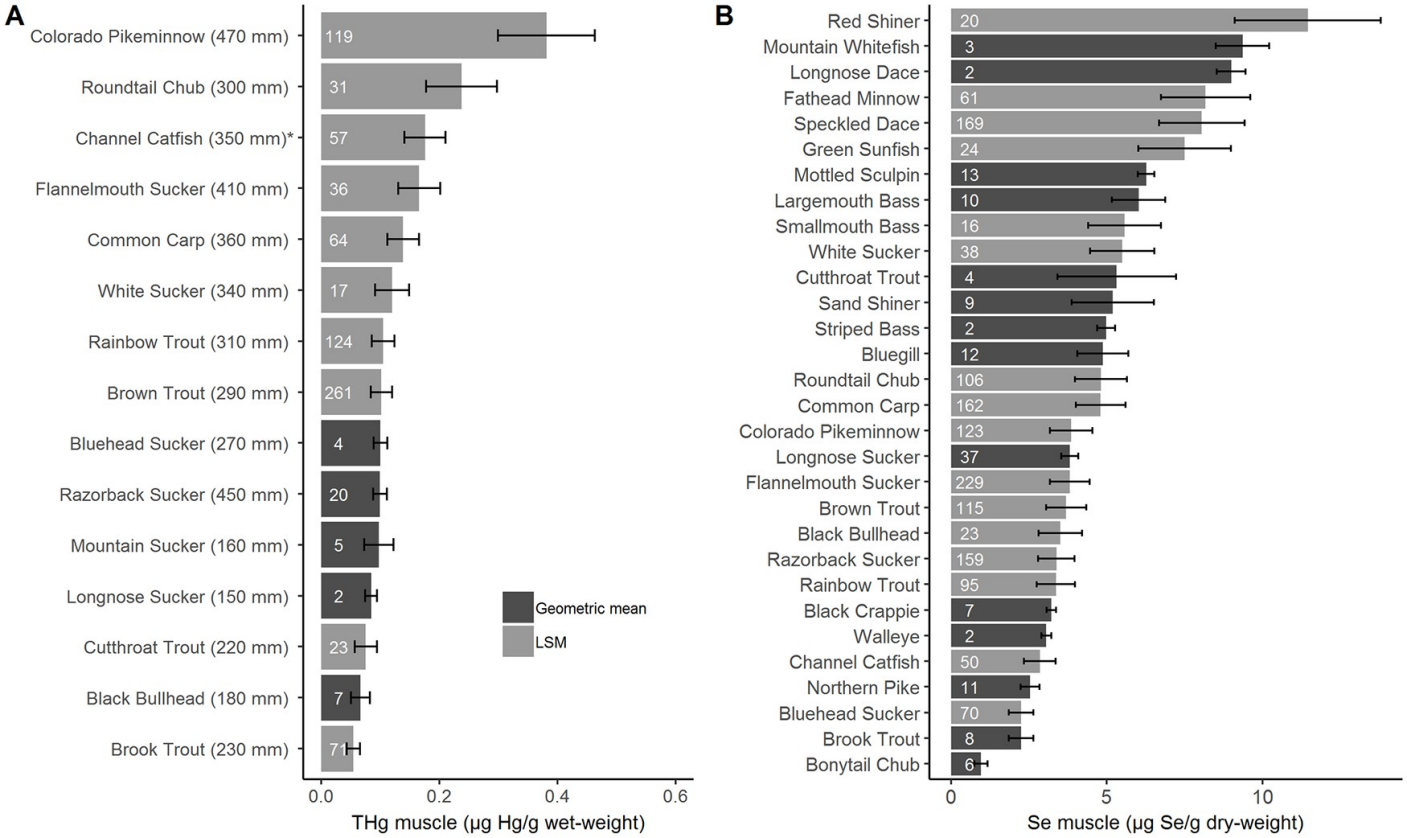
Mean total mercury (THg, Panel A) and selenium (Se, Panel B) concentrations in fish species of the Upper Colorado River Basin. **Least-squares mean concentrations represent the mean THg and Se concentration in each species after accounting for fish size (only for THg), tributary, site, and year effects using a mixed effects model (black shaded bars)**. THg concentrations in fish muscle were standardized to the respective median length of each species. An asterisk indicates there was no relationship between total length and THg; therefore, raw THg concentrations were used in the model. Geometric mean concentrations were calculated for species with insufficient spatial coverage to be included in the linear mixed effects models (< 15 individuals and/or found in < 3 tributaries; grey shaded bars). Mean total length (mm) of each species is in parenthesis for THg. White numbers indicate sample size. Error bars represent 1 standard error.

Seventeen of the 33 species in the database (52%) had individual exceedances of the THg-benchmark, including 13% (272/1959) of all individuals (S3 Table). This threshold was most frequently exceeded in Roundtail Chub (*Gila robusta*; 32% of all individuals, mean = 0.19 μg THg/g ww) and Colorado Pikeminnow (70% of individuals, mean = 0.35 μg THg/g ww). There were significant differences in THg among species after accounting for tributary, site, year, and fish size effects (Fig 2A and S4 Table; F_9, 486.49_ = 11.61; p<0.0001). Site accounted for 38% of the variance, while year accounted for 43% and 20% was unexplained by our model. Note that 5 species had insufficient data (see Methods) to be included in the linear mixed effects model and as a result are described by geometric means in the figure for visual comparisons but were not included in the pairwise comparison (Fig 2A and S4 Table). Least-squares mean THg concentrations were lowest in Brook Trout (*Salvelinus fontinalis*; size-adjusted THg = 0.06 ± 0.01 μg/g ww), and highest in Roundtail Chub (size-adjusted THg = 0.24 ± 0.06 μg/g ww) and Colorado Pikeminnow (size-adjusted THg = 0.38 ± 0.08 μg/g ww).

Eighty four percent of the species (27/32) in the database had individuals that exceeded the Se-benchmark including 48% (827 /1720) of all individuals sampled (S3 Table). Notable species include Speckled Dace (*Rhinichthys osculus*; 95% of individuals, mean = 9.75 μg Se/g dw), Roundtail Chub (66% of individuals, mean = 6.07 μg Se/g dw), and Colorado Pikeminnow (S3 Table; 63% of individuals, mean = 5.95 μg Se/g dw). There were significant differences in Se among species after accounting for tributary, site, year, and fish size effects (Fig 2B and S4 Table; F_15, 1393.5_ = 27.53; p<0.0001). Site accounted for 40% of the variance, year accounted for 16%, and 44% was left unexplained. In this case, 14 species could not be included in the linear mixed effects model due to insufficient data (see Methods) and are described by geometric means for visual comparisons (Fig 2B) but were not included in the pairwise comparison (S4 Table). Average Se concentrations in fish ranges from a low in Bonytail Chub (geometric mean = 0.96 μg/g dw) to a high in Red Shiner (*Cyprinella lutrensis*, Se = 11.45 μg/g dw), with other low trophic position species such as Fathead Minnow (*Pimephales promelas*), Speckled Dace, and Green Sunfish (*Lepomis cyanellus*) also having elevated Se concentrations (least-squares means = 8.17, 8.04, and 7.50 μg/g dw, respectively).

### THg and Se concentrations in fish assemblages by tributary

**Fig 3:**
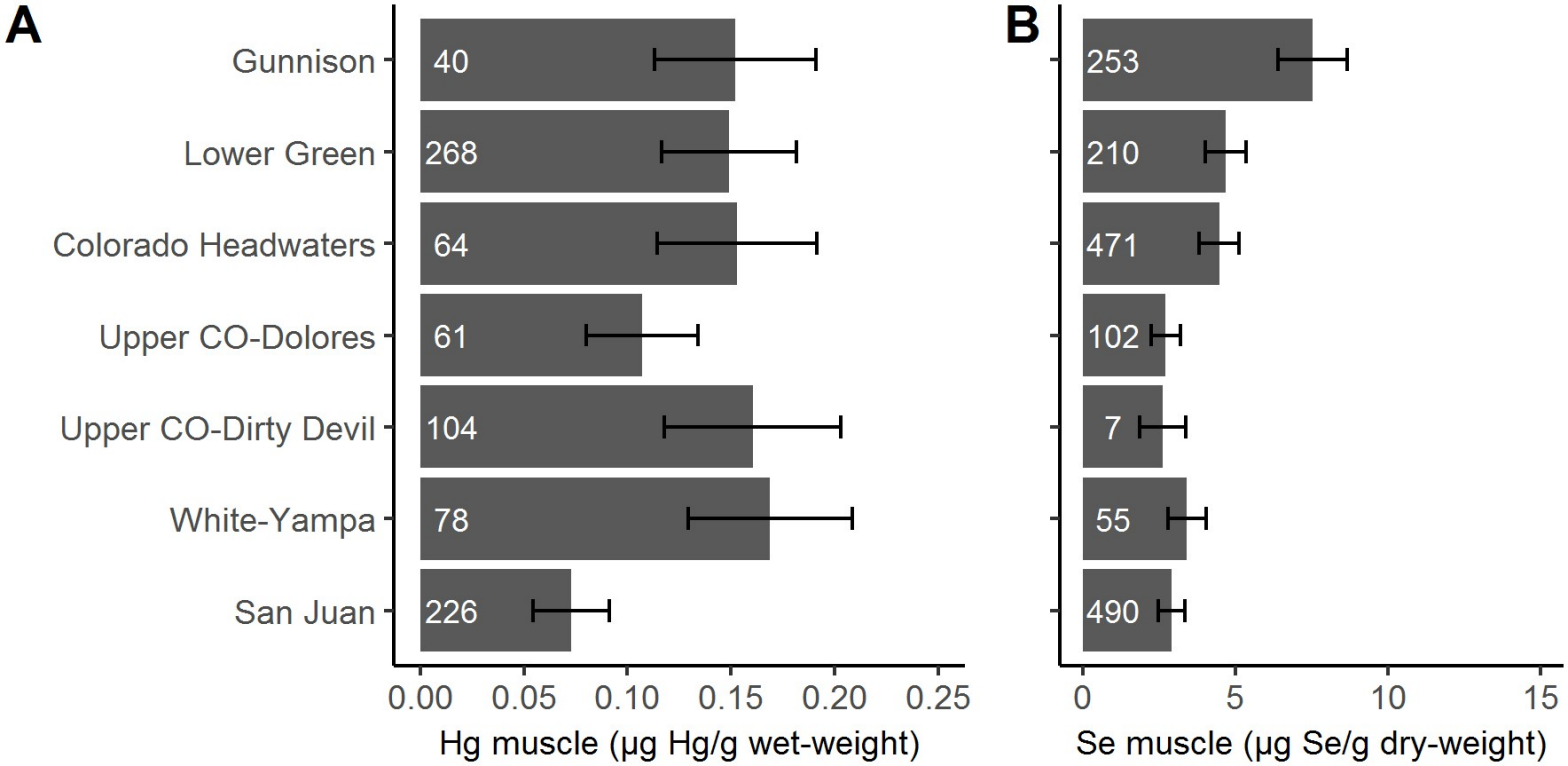
Least-squares means for total mercury (THg, size standardized, A) and selenium (Se, not size standardized, B) concentrations in muscle tissue from fish assemblages across tributaries of the Upper Colorado River Basin. Bars are least-squares means controlling for the effects of site, species, year, and size where appropriate. Error bars represent one standard error. White numbers indicate sample size.

Most THg exceedances were observed in the White-Yampa tributary (37% of all fish sampled) whereas the San Juan (4%) and Gunnison (3%) had the lowest number of exceedances (S5 Table). Least-squares mean THg concentrations of fish assemblages differed among tributaries of the UCRB after accounting for site, species, year, and fish size effects (Fig 3A and S6 Table; Tributary effect: F_6, 225.94_ = 6.02; p<0.001; Tributary/site effect: F_133, 607.71_ = 8.76; p<0.001). Species accounted for 29% of the variance in the data, year explained 52%, while 19% was left unexplained by our model. The fish assemblage in the White-Yampa had the highest THg concentrations (0.17 ± 0.04 μg/g wet-weight) while the San Juan had the lowest THg (0.07 ± 0.02 μg/g wet-weight). Most Se exceedances were observed in the Gunnison tributary (74%) while the San Juan (18%) had the lowest number of exceedances (S5 Table). Likewise, Se concentrations differed among tributaries after accounting for species, year, and site (Fig 3B and S6 Table; Tributary effect: F_6, 849.33_ = 3.07; p = 0.006; Tributary/site effect: F_144, 1346.82_ = 6.90; p<0.001). Species explained 52% of the variance in the data, year explained 36%, and 12% was left unexplained. The fish assemblages in the Gunnison (7.53 ± 1.12 ug/g dw), had the highest Se concentrations while the Dirty Devil had the lowest Se (2.61 ± 0.76 ug/g dw).

### Concentrations of THg and Se in adult Colorado Pikeminnow

Mercury concentrations in adult Colorado Pikeminnow varied 4-fold among tributaries, ranging from a low in the San Juan River (least-squares mean = 0.27 and standard error ± 0.01 μg/g ww), medium in the Colorado (0.44 ± 0.01 μg/g ww), and a high in the Green river (0.62 ± 0.01 μg/g ww). Selenium concentrations in adult Colorado Pikeminnow also varied 4-fold among tributaries, ranging from a low in the San Juan River (least-squares mean = 2.81 ± 0.09 μg/g ww), medium in the Colorado (6.51 ± 0.11 μg/g ww), and a high in the Green river (7.23 ± 0.18 μg/g ww).

### Se:THg molar ratios in fish species and assemblages by tributary

**Fig 4:**
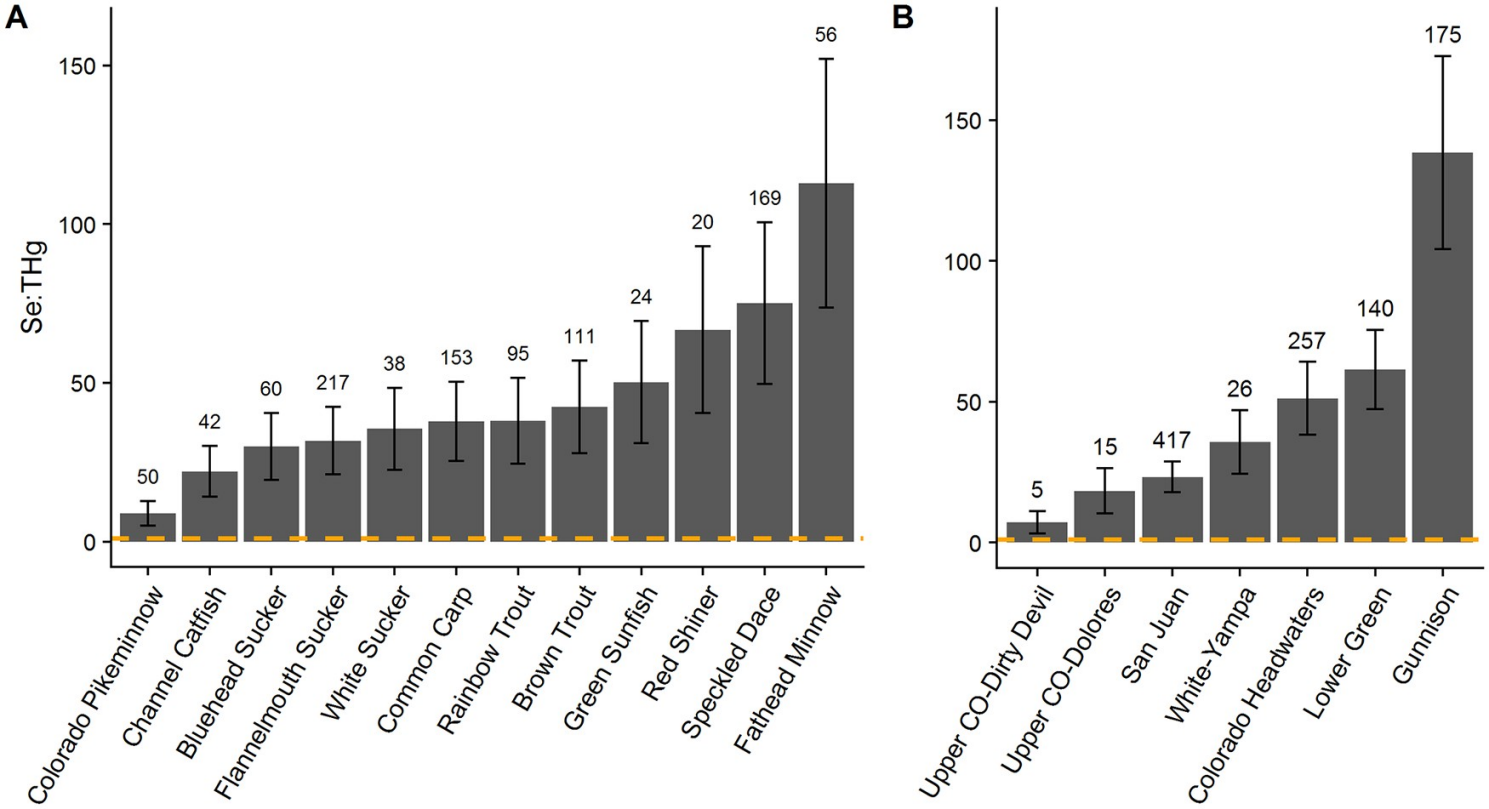
Least-squares means selenium: Mercury (Se:Hg) molar ratios of fish species (A) and assemblages (B) in the Upper Colorado River Basin. Bars are least-squares means controlling for the effects of site, tributary, and year (A) and site, species, and year (B), where appropriate. Error bars represent one standard error. Numbers above error bars indicate sample size. Dotted line represents a 1:1 molar ratio, which is thought to be protective of fish health.

Molar ratios of Se:THg differed among species (Fig 4A and S7 Table; F_11, 654.2_ = 12.65; p<0.0001) and all species were above 1 (a threshold shown to indicate potential protection from negative Hg consequences) [47]. Ratios ranged from 8.87 ± 3.9 in Colorado Pikeminnow to 112.87 ± 39.2 in Fathead Minnow. There were also differences in Se:THg molar ratios among tributaries (Fig 4B and S7 Table; Tributary effect: F_6,592.61_ = 4.98; p<0.0001; Tributary/site effect: F_126,788.65_ = 3.49; p<0.0001) after accounting for sites, species and year. The molar ratios of Se:Hg for each tributary were all above 1 ranging from 7.23 ± 3.97 in the Upper Colorado-Dirty Devil to 138.46 ± 34.28 in the Gunnison.

## Discussion

Concentrations of both THg and Se in UCRB fishes are above critical benchmarks in 13% and 48% of individual fish, respectively, suggesting the health of some fishes in the UCRB may be impaired by these contaminants. Among UCRB fishes, large long-lived native taxa have the highest THg concentrations. Two notable native species with some of the highest THg values are Colorado Pikeminnow and Roundtail Chub. Concentrations of Se are also elevated in many fishes of the UCRB but small species at lower trophic levels have the highest Se values in our dataset. In addition to taxonomic patterns of Hg and Se concentrations there are also spatial patterns in these data. While THg spatial patterns show similarity among the seven major drainage basins of the UCRB (only difference is the San Juan being lower than five basins), Se values show a pattern of elevated fish concentrations in three basins (Gunnison, Lower Green, and Colorado headwaters). While these elevated levels of THg and Se in fishes are noteworthy, it appears that Se:THg ratios for all species are above one, a ratio that has been purported to reduce the toxicity of MeHg [48–51].

Selenium concentrations in fish tissue are elevated above fish health standards throughout the UCRB while THg concentrations (relative to fish health benchmarks) are elevated in specific species of fish and exceed the fish health benchmark for THg more often in certain tributaries than others. Concentrations of Se and THg in fish assemblages among tributaries varied over a similar factor of about 2 but the range of absolute concentrations for Se in fish tissue are elevated relative to other western river basins whereas the THg concentrations observed in the UCRB are not uncommon to the western United States [52, 53]. Whereas for Hg, while some tributaries were found to have more frequent exceedances of fish health standards than others, absolute concentrations only exceeded fish health standards frequently in certain species.

Species of management concern frequently (30% of the time or more) exceeded a fish health standard for THg or Se (e.g., Flannelmouth Sucker-*Catostomus latipinnis*, Razorback Sucker) or were observed to exceed both standards more than 30% of the time (e.g., Roundtail Chub and Colorado Pikeminnow). Regarding Colorado Pikeminnow, even though few or no Pikeminnow in our database were from the tributaries with the highest THg (White-Yampa) or Se (Gunnison) in the fish assemblage analysis, this species was the only one with average concentrations in excess of both fish health standards. It is important to note that this study encompasses the entire native range of this species. These results combined with the continuing recent declines in Colorado Pikeminnow abundances [54–56], suggests that further understand of the current THg concentrations and their spatial patterns may prove useful in identifying additional drivers of declining temporal patterns in Colorado Pikeminnow abundance. Collectively, these findings support the idea that THg and Se are stressors that may be limiting the recovery of native fishes in the UCRB along with nonnative species, flow alteration, and habitat fragmentation [10, 16, 57].

### Spatial THg and Se concentrations

The disparity in spatial patterns between two contaminant (i.e., THg and Se) concentrations in fishes throughout the UCRB is likely a result not only of biotic factors (e.g., fish assemblage patterns and resulting variation in trophic dynamics) but also landscape and local abiotic factors. The UCRB likely has multiple sources of inorganic Hg, including an ongoing history of coal and hard rock mining, ongoing coal burning, and deposition from global sources [58–60]. However, it is ultimately landscape factors, such as land use, biogeochemistry, and habitat that result in localized conversion of inorganic Hg to MeHg, the most bioavailable form of Hg [53, 61]. Therefore, high inorganic Hg deposition in an area does not necessarily beget high MeHg production and accumulation, and remote areas, distanced from point sources of inorganic Hg, are also at risk to ecological harm from Hg [42, 53, 59]. As discussed in Eagles-Smith et al. [53], western North America, and subsequently the UCRB, contains a unique assemblage of habitats with drivers that promote MeHg production and transport including seasonal wetlands [58, 62], large river systems that traverse broad expanses of the region [63] and a network of reservoirs [61]. The seasonality of rivers in the West is characterized by pulsed hydrology, intermittent inundation, and short-term extreme ranges in primary productivity, all of which are linked to MeHg production [12].

Despite the widespread concern of Hg contamination of aquatic ecosystems and organisms, some tributaries in the UCRB have relatively little data, especially from native fish species. To prevent spurious comparisons among tributaries, using data from potentially disparate fish assemblages, we developed relativized estimates of fish THg concentrations. While this comparison proved useful for assessing relativized exposure across the riverscape, we remain limited in our abilities to understand what processes drive these patterns. Previous studies assessed the distribution of landscape variables relative to Hg in stream fish at regional and national scales in North America [9, 64, 65]; however, they are all comparatively broad in their spatial resolution. Alpers et al. [66] created a predictive model for Hg in fish in streams of the Sierra Nevada Mountains (California, USA) based on land cover/land use and the distribution of legacy gold mines. Such an approach may be applicable in the UCRB; however, this would require extensive sampling throughout the UCRB as well as a better understanding of how hydrology and water management affect localized biogeochemistry and subsequently MeHg production and assimilation.

While elevated Se concentrations have been reported in all tributaries of the UCRB, reviewed in [67, 68], areas of concern are those with extensive irrigation projects underlain by seleniferous soils which are rich in water soluble Se [69], including areas on the Green River, the San Juan River, the Gunnison River and the Grand Valley of the Colorado River. A study investigating irrigation water quality found that areas on the Green, Gunnison, and the headwaters of the Colorado rivers ranked among the highest for concentrations of Se in fish, sediments or water among 26 other reaches in the Western US [70]. Our model predictions of Se concentrations across fish assemblages corroborate these findings. Similar to THg, location accounted for 40% of the variance in the Se data, emphasizing the influence of landscape heterogeneity on Se concentrations in fish.

Areas with low flow and evaporation, such as wetlands, backwater ponds and reservoirs where irrigation canals terminate can be hotspots for production of more bioavailable forms of Se and accumulation in fish [70]. Given a large proportion of Colorado Pikeminnow and Razorback Sucker are found in the Lower Green River and headwaters of the Colorado River and that Se concentrations were relatively high in these fish assemblages, understanding drivers of Se accumulation in these river reaches could be important for the conservation of these endangered fishes. An example of such site-specific knowledge includes that Colorado Pikeminnow and Razorback Sucker use backwater pond areas as important habitat where fish tissue Se concentrations can be 2–3 times higher than those in the mainstem of the river, but local-scale factors that control Se accumulation into these fish remain unresolved [32, 34, 71–73]. While there are extensive programs (e.g., TMDL programs, salinity control programs, Gunnison Selenium Management Team) working to mitigate Se loading to rivers, less is known about how these efforts relate to Se in fish.

### Native fish vulnerability to accumulation of THg and Se

We found that two native fish species, Colorado Pikeminnow and Roundtail Chub, had the highest concentrations of THg after accounting for the effects of tributary, site, and sampling year, and modeling to the median respective lengths in our database for each species (490 mm and 300 mm; Fig 2A). The Colorado Pikeminnow is a long-lived piscivore at the top of the food chain within the UCRB [28] and their diet consists of almost entirely of other fishes once they exceed 200 mm [74]. Despite being smaller and less piscivorous than Colorado Pikeminnow, Roundtail Chub are also relatively long-lived [74] compared to other species, which may be the most influential factor explaining fish THg concentrations in the UCRB. The geometric mean Se concentration of Colorado Pikeminnow (used to compare raw concentrations against benchmarks values) exceeded the Se-benchmark (including 63% of all individuals). In addition, 35% of Razorback Sucker individuals, another endangered native species, exceeded the Se-benchmark, suggesting that some of the highly imperiled native fish fauna of the UCRB, in addition to being at risk to other more well-known stressors (i.e., streamflow alteration and nonnative species) are accumulating Hg and Se to levels believed to cause harm to fish.

The UCRB Recovery Program is tasked with restoring populations of endangered native fishes to sustainable levels while allowing for continued development of water resources [13, 14, 28]. One goal is to support Colorado Pikeminnow populations with an average size of 450 mm (a Colorado Pikeminnow is generally considered an adult once it reaches 450 mm) [56]. We estimated THg concentrations of Colorado Pikeminnow at 450 mm in each of the major tributaries where they are found. Based on the relationship between total length and THg concentrations of the 119 Colorado Pikeminnow (with lengths) in our database, adult fish exceeded the THg-benchmark in the Colorado (61% of individuals) and Green river populations (44% of individuals), while the San Juan population met the THg-benchmark (0.27 ug THg/g). Thus, risks to Colorado Pikeminnow vary among the three major tributaries, likely due to differences in size, age structure, and management practices (stocking of age-0 fish in San Juan) [75]. Thus, in the San Juan, fish Hg concentrations may not reflect the true risks to this populations. See Osmundson and Lusk [76] for a more thorough assessment of Hg risks to Colorado Pikeminnow and Roundtail Chub.

Like Hg, dietary exposure of fish to Se is the dominant pathway of uptake [77–81]. However, complexities arise when predicting Se concentrations in fish because speciation of Se and its availability to fish is sensitive to local biogeochemical conditions and bioaccumulation factors differ across trophic levels. The greatest bioaccumulation of Se occurs at the base of the food web, between periphyton, plankton, invertebrates and small-bodied fish, and the trophic transfer efficiency can decrease (trophic transfer function < 1) as you increase in trophic position [21, 82]. Therefore, Se concentrations in high-trophic-level fishes might be less than that of their potential prey items [34, 80, 83]. Among all species, we found that small-bodied fish have the highest Se concentrations across the UCRB (Fig 2B). While most small-bodied fish were high in Se, there were no other trophic-based patterns explaining Se concentrations (i.e., piscivorous Colorado Pikeminnow had similar Se concentrations to benthic-feeding Flannelmouth Sucker; Fig 2B). Differences in Se bioaccumulation are thought to be more related with food source than trophic position but could also be related to differences in reproductive strategies and depuration rates among species [8, 32, 84].

Unlike Hg, fish can depurate Se from their tissues. A particularly important depuration process is spawning; Se is transferred from female fish to their eggs and the amount partitioned differs among species and seasonal fluctuations of Se concentrations associated with spawning events [33, 79, 85]. Spawning frequency and lifespan also affect rates of Se depuration in fish [71, 86]. Fractional spawners, fish who spawn several times a year, have multiple opportunities for Se removal while fish that do not consistently spawn annually (i.e., skip spawners), such as the long-lived cyprinids (i.e., fish in the Cyprinidae family) native to Colorado River, have less opportunity for removal of Se through spawning [30, 87]. Such complex interactions between contaminant biodynamics and life history may partially explain the differences we observed between Se concentrations of native and non-native fishes of similar trophic positions. Finally, several others have speculated that Pikeminnow may not be efficient at depurating Se, retaining elevated concentrations in muscle tissue for years following events that seemed to decrease exposure [32, 80, 88].

### Se:THg molar ratios

There is some evidence that an excess of Se relative to Hg confers a protective advantage in fish. Selenium has a high binding affinity for Hg and MeHg which leads to the formation of permanently biologically unavailable Se-Hg precipitates. Ganther et al. [47] have suggested that a Se:Hg molar ratio above 1 is largely protective for the adverse effects of Hg and that risk assessments should consider Se:Hg ratios rather than Hg tissue concentrations alone. However, there is still tremendous uncertainty regarding the efficacy of Se for reducing Hg toxicity. In fact, recent studies indicate that Hg toxicity to some endpoints and some life stages is not influenced [89, 90] or even exacerbated by Se exposure [91, 92]. Therefore, further research is needed in order to better understand how the Se:Hg ratios may influence risk in UCRB fishes. Additionally, while Se may reduce Hg toxicity, Se itself can be toxic in excess, and if too much Se is bound to Hg, it is also possible for organisms to suffer from Se deficiency [93]. It is unclear whether excess Se (above toxicity benchmarks) can be mitigated by Hg exposure.

We found mean Se:THg molar ratios to be positive and above 1 in all species, concurrent with previous studies investigating Se:Hg molar ratios in freshwater fish (Fig 4A) [24, 25, 94, 95] though our ratios were much higher than other comparable species [24, 25]. The higher values are presumably a result of the elevated Se concentrations found across the UCRB. Considering 48% of individual fish in this study exceed Se toxicity thresholds, the issue of Se toxicity must be considered, while Se deficiency is unlikely. This is especially true of large bodied, piscivorous fish like the Colorado Pikeminnow, a finding supported by Peterson et al. [24] who found that three other species of Pikeminnow (Northern Pikeminnow-*Ptychocheilus oregonensis*, Sacramento Pikeminnow-*Ptychocheilus grandis*, and Umpqua Pikeminnow-*Ptychocheilus umpquae)* from 7 sites across the Northwestern US had Se:Hg values < 1. We saw similar patterns across fish assemblages (but not for Colorado Pikeminnow which had Se:Hg > 1). Assemblages with higher Se values tended to have greater protection from THg in our dataset. However, while it is important to consider the effect of Se and Hg together when assessing risks to fish health, the actual Se:Hg molar ratio protective of fish health remains unclear.

## Conclusions

Our results shed light on the potential risk of Hg and Se exposure for fishes of the UCRB and we have identified both data and knowledge gaps to address short comings of this work. Despite spanning 50 years of data collection, we still lack sufficient THg data on many native species found in the UCRB, including the Razorback Sucker and Bluehead Sucker. The dataset also does not reflect the most recent distribution of nonnative populations as there are little to no data from Smallmouth Bass (*Micropterus dolomieu*), Northern Pike (*Esox Lucius*) or Walleye (*Sander vitreus*). To address the risks that Se and Hg pose to the fish of the UCRB, a comprehensive survey of fish tissues would be necessary (no new data from tissues have been published since 2012) and mechanistic studies that examine the drivers of Hg and Se concentrations throughout the UCRB basin, and the spatial juxtaposition of contaminant hotspots to important fish habitats, could provide resource management with tools to help mitigate these threats.

## Supporting information

S1 Table Species and family specific conversion values used to convert between selenium wet-weight whole body concentrations to selenium wet-weight muscle plug. (DOCX)

S2 Table Species groupings, size distribution (total length), and total mercury concentrations (THg μg/g ww) in muscle tissue of 27 species of fish from Upper Colorado River Basin collected between 1962–2011. (DOCX)

S3 Table Total mercury (μg THg/g wet-weight) and selenium (μg Se/g dry-weight) concentrations in fish muscle tissue in 33 species of fish from Upper Colorado River Basin collected between 1962–2011. (DOCX)

S4 Table Least-squares mean, size standardized tissue total mercury (THg μg/g ww) and selenium (Se μg/g dw) concentrations in fish species of the Upper Colorado River Basin. (DOCX)

S5 Table Frequency that fish exceed fish health benchmarks for THg (0.27 ug Hg/g wet-weight) and Se (5.1 ug Se/g dw) and range of concentrations observed in each tributary of the Upper Colorado River Basin. (DOCX)

S6 Table Least-squares mean, size standardized tissue total mercury (THg μg/g ww) and selenium (Se μg/g dw) concentrations in fish assemblages of the Upper Colorado River Basin. (DOCX)

S7 Table Least-squares mean, selenium: Mercury (Se:THg) molar ratios in fish species and fish assemblages of the Upper Colorado River Basin. (DOCX)
