# Supplementary material for: Mercury and selenium concentrations in fishes of the Upper Colorado River Basin, southwestern United States: A retrospective assessment
Source: PLoS One. 2020 Jan 13;15(1):e0226824. doi: 10.1371/journal.pone.0226824 (PMC6957192; doi:10.1371/journal.pone.0226824)
Supplement: S1 Table — (DOCX) [file pone.0226824.s001.docx]

| **S1 Table.** **Species and family specific conversion values used to convert between Selenium wet-weight whole body concentrations to Selenium wet-weight muscle plug.**^38^ | | |
| --- | --- | --- |
| Species | M/WB | M/WB Source |
| Black Bullhead | 1.27 | All fish |
| Black Crappie | 1.23 | Centrarchidae |
| Bluegill | 1.23 | Centrarchidae |
| Bluehead Sucker | 1.34 | Catostomidae |
| Bonytail Chub | 1.33 | Cyprinidae |
| Brook Trout | 1.27 | Salmonidae |
| Brown Trout | 1.27 | Salmonidae |
| Channel Catfish | 1.27 | All fish |
| Colorado Pikeminnow | 1.33 | Cyprinidae |
| Common Carp | 1.33 | Cyprinidae |
| Cutthroat Trout | 1.27 | Salmonidae |
| Fathead Minnow | 1.33 | Cyprinidae |
| Flannelmouth Sucker | 1.46 | Flannelmouth Sucker |
| Green Sunfish | 1.23 | Green Sunfish |
| Largemouth Bass | 1.23 | Centrarchidae |
| Longnose Dace | 1.33 | Cyprinidae |
| Longnose Sucker | 1.34 | Catostomidae |
| Mottled Sculpin | 1.27 | All fish |
| Mountain Sucker | 1.34 | Catostomidae |
| Mountain Whitefish | 1.27 | Salmonidae |
| Northern Pike | 1.27 | All fish |
| Rainbow Trout | 1.27 | Salmonidae |
| Razorback Sucker | 1.34 | Catostomidae |
| Red Shiner | 1.33 | Cyprinidae |
| Roundtail Chub | 1.05 | Roundtail Chub |
| Sand Shiner | 1.33 | Cyprinidae |
| Smallmouth Bass | 1.23 | Smallmouth Bass |
| Speckled Dace | 1.33 | Cyprinidae |
| Striped Bass | 1.23 | Centrarchidae |
| Walleye | 1.23 | Percidae |
| White Sucker | 1.34 | White Sucker |
| Yellow Perch | 1.23 | Percidae |
| If species or family specific values were not available, we used the average value for all fish. M-Muscle, WB-Whole body.  Common Shiner are not included here because there are no Se data for this species | | |
