# Supplementary material for: Mercury and selenium concentrations in fishes of the Upper Colorado River Basin, southwestern United States: A retrospective assessment
Source: PLoS One. 2020 Jan 13;15(1):e0226824. doi: 10.1371/journal.pone.0226824 (PMC6957192; doi:10.1371/journal.pone.0226824)
Supplement: S2 Table — (DOCX) [file pone.0226824.s002.docx]

| **S2 Table. Species groupings, size distribution (total length), and total mercury concentrations (THg µg/g ww) in muscle tissue of 27 species of fish from Upper Colorado River Basin collected between 1962-2011.** | | | | | | |
| --- | --- | --- | --- | --- | --- | --- |
| Species Grouping | N-tributaries | Forage Guild | Species | N-total | Median TL (mm)  (Min-Max) | Median [THg] (µg/g) (Min-Max) |
| Catostomidae | 5 | Benthivore | Bluehead Sucker | 4 | 320 (85-340) | 0.09 (0.071-0.13) |
|  |  | Benthivore | Flannelmouth Sucker | 36 | 430 (210-540) | 0.13 (0.014-0.32) |
|  |  | Benthivore | Longnose Sucker | 2 | 150 (120-180) | 0.086 (0.072-0.1) |
|  |  | Benthivore | Mountain Sucker | 5 | 160 (140-180) | 0.099 (0.058-0.19) |
|  |  | Benthivore | Razorback Sucker | 20 | 440 (360-570) | 0.12 (0.042-0.24) |
|  |  | Benthivore | White Sucker | 19 | 320 (230-460) | 0.14 (0.024-0.41) |
| *CC | 5 | Benthivore | Common Carp | 72 | 390 (80-670) | 0.13 (0.011-0.38) |
| *Centrarchidae nonpiscivorous | 4 | Generalist | Bluegill | 6 | 150 (98-200) | 0.16 (0.1-0.31) |
|  |  | Generalist | Green Sunfish | 7 | 120 (49-140) | 0.14 (0.038-0.2) |
| *Centrarchidae  piscivorous | 5 | Generalist | Black Crappie | 3 | 160 (140-160) | 0.1 (0.1-0.1) |
|  |  | Piscivore | Black Crappie | 4 | 220 (200-270) | 0.2 (0.15-0.25) |
|  |  | Piscivore  Generalist if TL<140 | Largemouth Bass | 5 | 310 (110-400) | 0.1 (0.09-0.1) |
|  |  | Piscivore  Generalist if TL<150 | Smallmouth Bass | 12 | 240 (150-350) | 0.17 (0.048-0.38) |
| *Coregoninae | 2 | Generalist invertivore | Mountain Whitefish | 6 | 240 (220-370) | 0.06 (0.038-0.18) |
| *Cottidae | 1 | Generalist invertivore | Mottled Sculpin | 3 | 140 (140-140) | 0.055 (0.053-0.062) |
| CPM | 6 | Piscivore | Colorado Pikeminnow | 119 | 490 (220-820) | 0.55 (0.036-1.8) |
| *Cyprinidae small bodied | 1 | Generalist invertivore | Red Shiner | 1 | 62 (62-62) | 0.04 (0.04-0.04) |
|  |  | Generalist invertivore | Speckled Dace | 14 | 89 (70-160) | 0.097 (0.033-0.19) |
| *Esocidae | 1 | Piscivore | Northern Pike | 8 | 370 (220-820) | 0.11 (0.1-0.31) |
| Ictaluridae | 5 | Generalist | Black Bullhead | 7 | 160 (140-220) | 0.05 (0.033-0.14) |
|  |  | Generalist | Channel Catfish | 63 | 320 (150-630) | 0.2 (0.01-0.91) |
| *Moronidae | 1 | Piscivore | Striped Bass | 2 | 500 (410-590) | 0.22 (0.12-0.31) |
|  |  | Piscivore | Walleye | 2 | 450 (440-460) | 0.16 (0.13-0.18) |
| RTC | 3 | Piscivore | Roundtail Chub | 31 | 300 (210-430) | 0.28 (0.11-2) |
| Salmonidae | 7 | Generalist | Brook Trout | 71 | 210 (140-420) | 0.051 (0.02-0.28) |
|  |  | Generalist | Brown Trout | 263 | 290 (42-590) | 0.11 (0.016-0.86) |
|  |  | Generalist | Cutthroat Trout | 23 | 220 (160-290) | 0.072 (0.027-0.15) |
|  |  | Generalist | Rainbow Trout | 124 | 300 (130-510) | 0.053 (0.011-0.47) |
| * Species groups without length-THg relationships or insufficient spatial coverage (found in <3 tributaries and/or less than 25 individuals total) and thus not size standardized for statistical modeling. Species highlighted in grey are native to the Upper Colorado River Basin.  Species without length data and thus not included in size-correction analysis: Yellow Perch, Bonytail Chub, Common Shiner, Fathead Minnow, Longnose Dace, and San Shiner. | | | | | | |
