# Supplementary material for: Mercury and selenium concentrations in fishes of the Upper Colorado River Basin, southwestern United States: A retrospective assessment
Source: PLoS One. 2020 Jan 13;15(1):e0226824. doi: 10.1371/journal.pone.0226824 (PMC6957192; doi:10.1371/journal.pone.0226824)
Supplement: S3 Table — (DOCX) [file pone.0226824.s003.docx]

| **S3 Table. Total mercury (µg THg/g wet-weight) and selenium (µg Se/g dry-weight) concentrations in fish muscle tissue in 33 species of fish from Upper Colorado River Basin collected between 1962-2011.** | | | | | | | | | | | | | | |
| --- | --- | --- | --- | --- | --- | --- | --- | --- | --- | --- | --- | --- | --- | --- |
| Family | N Hg | % over  THg-mark | Mean Hg  (Min-Max) | N Se | % over Se-  mark | Mean Se  (Min-Max) | Species | N Hg | Native | % over  THg-mark | Mean Hg  (Min-Max) | N Se | % over  Se-mark | Mean Se  (Min-Max) |
| Catostomidae | 440 | 5 | 0.081 (0.001-0.506) | 595 | 33 | 4.213 (0.11-85.76) | Bluehead Sucker | 62 | Native | - | 0.052 (0.017-0.156) | 70 | 10 | 2.66 (1.033-12.06) |
|  |  |  |  |  |  |  | Flannelmouth Sucker | 226 | Native | 4 | 0.088 (0.013-0.36) | 229 | 31 | 3.796 (0.11-30.66) |
|  |  |  |  |  |  |  | Longnose Sucker | 39 | Non-native | - | 0.081 (0.031-0.196) | 37 | 27 | 3.815 (1.193-9.514) |
|  |  |  |  |  |  |  | Mountain Sucker | 6 | Native | - | 0.088 (0.051-0.193) | 1 | 100 | 16.08 (16.08-16.08) |
|  |  |  |  |  |  |  | Razorback Sucker | 62 | Native | 16 | 0.116 (0.042-0.506) | 159 | 35 | 4.513 (0.429-54.1) |
|  |  |  |  |  |  |  | White Sucker | 45 | Non-native | 4 | 0.059 (0.001-0.405) | 99 | 54 | 6.803 (1.501-85.76) |
| Centrarchidae | 75 | 9 | 0.095 (0.001-0.425) | 126 | 65 | 7.256 (2.417-123) | Black Crappie | 7 | Non-native | - | 0.146 (0.1-0.25) | 7 | - | 3.218 (2.417-3.708) |
|  |  |  |  |  |  |  | Bluegill | 9 | Non-native | 11 | 0.101 (0.039-0.31) | 12 | 42 | 4.876 (2.667-12.9) |
|  |  |  |  |  |  |  | Green Sunfish | 29 | Non-native | - | 0.05 (0.001-0.199) | 77 | 77 | 9.068 (2.608-123) |
|  |  |  |  |  |  |  | Largemouth Bass | 9 | Non-native | 11 | 0.109 (0.046-0.327) | 10 | 60 | 6.019 (3.625-11.316) |
|  |  |  |  |  |  |  | Smallmouth Bass | 19 | Non-native | 21 | 0.188 (0.048-0.425) | 18 | 61 | 5.783 (3.05-14.76) |
|  |  |  |  |  |  |  | Striped Bass | 2 | Non-native | 50 | 0.194 (0.121-0.309) | 2 | 50 | 4.977 (4.7-5.27) |
| Cottidae | 20 | - | 0.038 (0.016-0.142) | 13 | 92 | 6.26 (4.699-8.128) | Mottled Sculpin | 20 | Native | - | 0.038 (0.016-0.142) | 13 | 92 | 6.26 (4.699-8.128) |
| Cyprinidae | 703 | 20 | 0.108 (0.001-1.97) | 666 | 68 | 7.268 (0.443-146.3) | Bonytail Chub | 6 | Native | - | 0.106 (0.071-0.188) | 6 | - | 0.955 (0.694-2.012) |
|  |  |  |  |  |  |  | Colorado Pikeminnow | 132 | Native | 70 | 0.354 (0.036-1.83) | 123 | 63 | 5.949 (0.443-30.7) |
|  |  |  |  |  |  |  | Common Carp | 173 | Non-native | 3 | 0.09 (0.009-0.378) | 170 | 49 | 6.028 (1.729-110.39) |
|  |  |  |  |  |  |  | Common Shiner | 1 | Non-native | - | 0.136 (0.136-0.136) | - | - | - |
|  |  |  |  |  |  |  | Fathead Minnow | 56 | Non-native | - | 0.031 (0.003-0.163) | 61 | 67 | 11.891 (1.596-146.3) |
|  |  |  |  |  |  |  | Longnose Dace | 2 | Non-native | - | 0.079 (0.072-0.086) | 2 | 100 | 8.992 (8.539-9.47) |
|  |  |  |  |  |  |  | Red Shiner | 20 | Non-native | 5 | 0.032 (0.003-0.27) | 20 | 75 | 12.382 (3.086-115.71) |
|  |  |  |  |  |  |  | Roundtail Chub | 128 | Native | 32 | 0.191 (0.042-1.97) | 106 | 66 | 6.067 (2.31-19.95) |
|  |  |  |  |  |  |  | Sand Shiner | 9 | Non-native | - | 0.048 (0.012-0.133) | 9 | 33 | 5.188 (3.059-15.504) |
|  |  |  |  |  |  |  | Speckled Dace | 176 | Native | 1 | 0.061 (0.001-0.461) | 169 | 95 | 9.752 (3.059-50.54) |
| Esocidae | 11 | 18 | 0.168 (0.1-0.416) | 11 | - | 2.538 (1.708-4.572) | Northern Pike | 11 | Non-native | 18 | 0.168 (0.100-0.416) | 11 | - | 2.538 (1.708-4.572) |
| Ictaluridae | 106 | 18 | 0.132 (0.01-0.91) | 79 | 22 | 3.514 (1.49-13.589) | Black Bullhead | 13 | Non-native | - | 0.052 (0.029-0.135) | 23 | 39 | 4.351 (1.535-9.538) |
|  |  |  |  |  |  |  | Channel Catfish | 93 | Non-native | 20 | 0.151 (0.01-0.91) | 56 | 14 | 3.22 (1.49-13.589) |
| Percidae | 4 | - | 0.093 (0.047-0.184) | 3 | 33 | 5.018 (2.9-13.53) | Walleye | 2 | Non-native | - | 0.156 (0.132-0.184) | 2 | - | 3.056 (2.9-3.22) |
|  |  |  |  |  |  |  | Yellow Perch | 2 | Non-native | - | 0.056 (0.047-0.066) | 1 | 100 | 13.53 (13.53-13.53) |
| Salmonidae | 600 | 14 | 0.084 (0.01-0.862) | 227 | 29 | 3.709 (0.875-31.75) | Brook Trout | 80 | Non-native | 4 | 0.06 (0.02-0.322) | 8 | - | 2.242 (0.875-4.175) |
|  |  |  |  |  |  |  | Brown Trout | 332 | Non-native | 23 | 0.11 (0.01-0.862) | 117 | 32 | 3.986 (1.194-31.75) |
|  |  |  |  |  |  |  | Cutthroat Trout | 28 | Native | - | 0.06 (0.018-0.147) | 4 | 50 | 5.318 (2.398-11.188) |
|  |  |  |  |  |  |  | Mountain Whitefish | 7 | Native | - | 0.064 (0.038-0.185) | 3 | 100 | 9.356 (8.139-11.063) |
|  |  |  |  |  |  |  | Rainbow Trout | 153 | Non-native | 3 | 0.059 (0.011-0.466) | 95 | 24 | 3.387 (0.974-11.049) |
| % over THg-mark and % over THg-mark represent the number of individuals that exceed the THg-benchmark = 0.27 ug THg/g and Se-benchmark= 5.1 ug Se/g dw. | | | | | | | | | | | | | | |
