# Supplementary material for: Mercury and selenium concentrations in fishes of the Upper Colorado River Basin, southwestern United States: A retrospective assessment
Source: PLoS One. 2020 Jan 13;15(1):e0226824. doi: 10.1371/journal.pone.0226824 (PMC6957192; doi:10.1371/journal.pone.0226824)
Supplement: S4 Table — (DOCX) [file pone.0226824.s004.docx]

| **S4 Table. Least-squares mean, size standardized tissue total mercury (THg µg/g ww) and selenium (Se µg/g dw) concentrations in fish species of the Upper Colorado River Basin.** | | | | |
| --- | --- | --- | --- | --- |
| Common name | Mean [THg] (µg/g) | Standard Error [THg] (µg/g) | N | Pairwise Comparison |
| Colorado Pikeminnow | 0.381 | 0.082 | 119 | E |
| Roundtail Chub | 0.238 | 0.060 | 31 | DE |
| *Channel Catfish | 0.176 | 0.035 | 57 | D |
| Flannelmouth Sucker | 0.166 | 0.036 | 36 | CD |
| Common Carp | 0.139 | 0.027 | 64 | BCD |
| White Sucker | 0.120 | 0.029 | 17 | BCD |
| Rainbow Trout | 0.105 | 0.019 | 124 | BC |
| Brown Trout | 0.102 | 0.018 | 261 | BC |
| Cutthroat Trout | 0.076 | 0.019 | 23 | AB |
| Brook Trout | 0.055 | 0.011 | 71 | A |
|  |  |  |  |  |
| Common name | Mean [Se] (µg/g) | Standard Error [Se] (µg/g) | N | Pairwise Comparison |
| Red Shiner | 11.450 | 2.345 | 20 | H |
| Fathead Minnow | 8.167 | 1.431 | 61 | GH |
| Speckled Dace | 8.046 | 1.372 | 169 | GH |
| Green Sunfish | 7.499 | 1.488 | 24 | FGH |
| Smallmouth Bass | 5.574 | 1.170 | 16 | CDEFG |
| White Sucker | 5.495 | 1.032 | 38 | EFG |
| Roundtail Chub | 4.817 | 0.832 | 106 | DEF |
| Common Carp | 4.803 | 0.797 | 162 | DEF |
| Colorado Pikeminnow | 3.861 | 0.680 | 123 | BCDE |
| Flannelmouth Sucker | 3.812 | 0.632 | 229 | BCDE |
| Brown Trout | 3.693 | 0.649 | 115 | BCDE |
| Black Bullhead | 3.504 | 0.697 | 23 | ABCDE |
| Razorback Sucker | 3.386 | 0.584 | 159 | BC |
| Rainbow Trout | 3.365 | 0.618 | 95 | ABCD |
| Channel Catfish | 2.849 | 0.507 | 50 | AB |
| Bluehead Sucker | 2.244 | 0.392 | 70 | A |
| Data represent species with a total sample size of >15 individuals and who were found in ≥3 tributaries. Least-squares mean concentrations represent the mean THg and Se concentration in each species after accounting for fish size (only for THg), tributary, site, and year effects using a mixed effects model. *Species without total length and THg relationship; therefore, raw THg concentrations were used in the model. Pairwise comparison based on α=0.05  -The following species were not included the THg least-square means modeling: Species without length data and thus not included in size-correction analysis: Yellow Perch, Bonytail Chub, Common Shiner, Fathead Minnow, Longnose Dace, and San Shiner; Species with a total sample size of <15 individuals or who were found in<3 tributaries: Bluehead Sucker, Longnose Sucker, Mountain Sucker, Razorback Sucker, Bluegill, Green Sunfish, Black Crappie, Largemouth Bass, Smallmouth Bass, Mountain Whitefish, Mottled Sculpin, Red Shiner, Speckled Dace, Northern Pike, Black Bullhead, Striped Bass, Walleye. | | | | |
