# Supplementary material for: Mercury and selenium concentrations in fishes of the Upper Colorado River Basin, southwestern United States: A retrospective assessment
Source: PLoS One. 2020 Jan 13;15(1):e0226824. doi: 10.1371/journal.pone.0226824 (PMC6957192; doi:10.1371/journal.pone.0226824)
Supplement: S5 Table — (DOCX) [file pone.0226824.s005.docx]

| **S5 Table. Frequency that fish exceed fish health benchmarks for THg (0.27 ug Hg/g wet-weight) and Se (5.1 ug Se/g dw) and range of concentrations observed in each tributary of the Upper Colorado River Basin.** | | | | | | |
| --- | --- | --- | --- | --- | --- | --- |
| Tributary | N Hg | % over  THg-benchmark | (Min-Max) | N Se | % over  Se-benchmark | (Min-Max) |
| Colorado Headwaters | 405 | 8 | (0.001-0.81) | 471 | 59 | (0.69-30.7) |
| Gunnison | 291 | 3 | (0.001-1.66) | 257 | 74 | (1.74-146.3) |
| Lower Green | 442 | 26 | (0.003-1.08) | 218 | 56 | (0.43-133) |
| San Juan | 490 | 4 | (0.011-0.47) | 494 | 18 | (0.11-31.92) |
| Upper CO-Dirty Devil | 113 | 31 | (0.023-0.8) | 7 | 29 | (2.12-6.97) |
| Upper CO-Dolores | 95 | 17 | (0.018-0.99) | 214 | 60 | (1.47-28.1) |
| White-Yampa | 123 | 37 | (0.025-1.97) | 59 | 31 | (0.88-11.06) |
