# Supplementary material for: Mercury and selenium concentrations in fishes of the Upper Colorado River Basin, southwestern United States: A retrospective assessment
Source: PLoS One. 2020 Jan 13;15(1):e0226824. doi: 10.1371/journal.pone.0226824 (PMC6957192; doi:10.1371/journal.pone.0226824)
Supplement: S6 Table — (DOCX) [file pone.0226824.s006.docx]

| **S6 Table. Least-squares mean, size standardized tissue total mercury (THg µg/g ww) and selenium (Se µg/g dw) concentrations in fish assemblages of the Upper Colorado River Basin**. | | | | |
| --- | --- | --- | --- | --- |
| Tributary | Mean [THg] (µg/g) | Standard Error  [THg] (µg/g) | N | Pairwise Comparison |
| Gunnison | 0.152 | 0.039 | 40 | ABC |
| Lower Green | 0.149 | 0.033 | 268 | BC |
| Colorado Headwaters | 0.153 | 0.038 | 64 | BC |
| Upper CO-Dolores | 0.107 | 0.027 | 61 | AB |
| Upper CO-Dirty Devil | 0.160 | 0.042 | 104 | C |
| White-Yampa | 0.169 | 0.039 | 78 | BC |
| San Juan | 0.073 | 0.018 | 226 | A |
| Tributary | Mean [Se] (µg/g) | Standard Error  [Se] (µg/g) | N | Pairwise Comparison |
| Gunnison | 7.532 | 1.124 | 253 | C |
| Lower Green | 4.688 | 0.671 | 210 | B |
| Colorado Headwaters | 4.465 | 0.658 | 471 | B |
| Upper CO-Dolores | 2.709 | 0.483 | 102 | A |
| Upper CO-Dirty Devil | 2.605 | 0.755 | 7 | AB |
| White-Yampa | 3.411 | 0.619 | 55 | AB |
| San Juan | 2.913 | 0.428 | 490 | A |
| Least-squares mean concentrations represent the mean THg and Se concentration in each tributary after accounting for fish size (only for THg), species, site, and year effects using a mixed effects model. Pairwise comparison based on α=0.05. | | | | |
