# Supplementary material for: Mercury and selenium concentrations in fishes of the Upper Colorado River Basin, southwestern United States: A retrospective assessment
Source: PLoS One. 2020 Jan 13;15(1):e0226824. doi: 10.1371/journal.pone.0226824 (PMC6957192; doi:10.1371/journal.pone.0226824)
Supplement: S7 Table — (DOCX) [file pone.0226824.s007.docx]

| **S7 Table. Least-squares mean, Selenium: Mercury (Se:THg) molar ratios in fish species and fish assemblages of the Upper Colorado River Basin.** | | | | |
| --- | --- | --- | --- | --- |
| Common name | Mean Se:Hg | Standard Error Se:Hg | N | Pairwise Comparison |
| Colorado Pikeminnow | 8.875 | 3.886 | 50 | A |
| Channel Catfish | 22.153 | 7.926 | 42 | AB |
| Bluehead Sucker | 30.023 | 10.519 | 60 | ABC |
| Flannelmouth Sucker | 31.816 | 10.562 | 217 | ABC |
| White Sucker | 35.506 | 12.894 | 38 | ABC |
| Common Carp | 37.864 | 12.526 | 153 | BC |
| Rainbow Trout | 38.093 | 13.557 | 95 | BC |
| Brown Trout | 42.457 | 14.526 | 111 | BCD |
| Green Sunfish | 50.204 | 19.234 | 24 | BCDE |
| Red Shiner | 66.751 | 26.286 | 20 | CDE |
| Speckled Dace | 75.117 | 25.443 | 169 | DE |
| Fathead Minnow | 112.869 | 39.210 | 56 | E |
| Subbregion | Mean Se:Hg | Standard Error Se:Hg | N | Pairwise Comparison |
| Upper CO-Dirty Devil | 7.233 | 3.971 | 5 | A |
| Upper CO-Dolores | 18.363 | 8.071 | 15 | AB |
| San Juan | 23.318 | 5.515 | 417 | A |
| White-Yampa | 35.709 | 11.355 | 26 | ABC |
| Colorado Headwaters | 51.195 | 12.909 | 257 | BC |
| Lower Green | 61.487 | 14.073 | 140 | C |
| Gunnison | 138.462 | 34.288 | 175 | D |
| Data represent species with a total sample size of >15 individuals and who were found in ≥3 tributaries. Least-squares mean concentrations of species represent the mean Se:Hg molar ratio after accounting for tributary, site, and year effects using a mixed effects model. Least-squares mean concentrations of tributaries represent the mean Se:Hg molar ratio of the fish assemblage found in each tributary after accounting for site, species, and year effects using a mixed effects model. Pairwise comparison based on α=0.05.  -The following species were not included in the least-squares means modeling of Se:Hg molar ratios byspecies: Species without Se:THg molar ratios Black Crappie, Common Shiner; Species with <15 individuals and/or found in <3 tributaries: Longnose Sucker, Mountain Sucker, Razorback Sucker, Black Crappie, Bluegill, Green Sunfish, Largemouth Bass, Smallmouth Bass, Striped Bass, Mottled Sculpin, Bonytail Chub, Common Shiner, Fathead Minnow, Longnose Dace, Roundtail Chub, Sand Shiner, Northern Pike, Black Bullhead, Walleye, Yellow Perch, Brook Trout, Cutthroat Trout, Mountain Whitefish. | | | | |
|  | | | | |
